# Correlations between Diabetes Mellitus Self-Care Activities and Glycaemic Control in the Adult Population: A Cross-Sectional Study

**DOI:** 10.3390/healthcare10010174

**Published:** 2022-01-17

**Authors:** Mihaela Simona Popoviciu, Violeta Nicoleta Marin, Cosmin Mihai Vesa, Simona Diana Stefan, Roxana Adriana Stoica, Cristian Serafinceanu, Emanuele Maria Merlo, Ali A Rizvi, Manfredi Rizzo, Stefan Busnatu, Anca Pantea Stoian

**Affiliations:** 1 Department of Internal Medicine II, Diabetes Mellitus, Clinical County Emergency Hospital of Oradea, 410167 Oradea, Romania; elapopoviciu@yahoo.com (M.S.P.); v_cosmin_15@yahoo.com (C.M.V.); 2 Department of Medical Disciplines, Faculty of Medicine and Pharmacy, University of Oradea, 410087 Oradea, Romania; 3 Department of Diabetes, Nutrition and Metabolic Diseases, “Carol Davila” University of Medicine and Pharmacy, 020021 Bucharest, Romania; violeta_marin95@yahoo.com (V.N.M.); simona_ds2002@yahoo.com (S.D.S.); criserafinceanu@gmail.com (C.S.); manfredi.rizzo@unipa.it (M.R.); ancastoian@yahoo.com (A.P.S.); 4 National Institute of Diabetes, Nutrition and Metabolic Diseases, “Prof N.C. Paulescu”, 020475 Bucharest, Romania; 5 Department of Human and Pediatric Pathology “Gaetano Barresi”, University of Messina, 98122 Messina, Italy; emanuelemaria.merlo@unime.it; 6 Department of Medicine, College of Medicine, University of Central Florida, Orlando, FL 32827, USA; Ali.Rizvi@UCF.edu; 7 Division of Endocrinology, Diabetes and Metabolism, School of Medicine, University of South Carolina, Columbia, SC 29209, USA; 8 Department of Health Promotion, Mother and Child Care, Internal Medicine and Medical Specialties (Promise), School of Medicine, University of Palermo, 90133 Palermo, Italy; 9 Department of Cardiology, “Carol Davila” University of Medicine and Pharmacy, 050474 Bucharest, Romania

**Keywords:** diabetes mellitus, diabetes self-management questionnaire, physical activity, self-care activities, glycaemic control, prevention

## Abstract

Although it is well known that lifestyle changes can affect plasma glucose levels, there is little formal evidence for the sustained effectiveness of exercise and diet in diabetes mellitus (DM) management. Self-care in DM refers to the real-life application of the knowledge that the patient gained during the education programmes. The goals are to bring about changes in the patient’s behaviour, thus improving glycaemic control. We evaluated the influence of DM self-care activities (SCA) on glycaemic control in a total of 159 patients with DM. Plasma glycated haemoglobin (HbA1c) levels were used to monitor glycaemic control, while SCA were assessed using the standardised Diabetes Self-Management Questionnaire (DSMQ). In our study, 53% of the patients had a HbA1c ≥ 7%. In univariate linear regression models, a statistically significant inverse association was observed between the HbA1c (the dependent variable) and both the DSMQ Dietary Control Score (R2 = 0.037, *p* = 0.0145) and the DSMQ Sum Score (R2 = 0.06, *p* = 0.0014). The mean absolute change in the HbA1c% associated with one standard deviation (SD) change in the DSMQ Sum Score, independent of the other significant variables retained in the compacted multivariate regression model, was −0.419% (confidence interval: 95%: from −0.18 to −0.65). Although the impact of the DSMQ Score was modest when compared to the other independent variables in the multivariate model, the findings emphasise the importance of maintaining optimal lifestyle changes to avoid hyperglycaemia and its complications. In conclusion, enhanced self-management of DM is associated with improved glucose control. In patients with chronic diseases such as DM, the role of streamlining SCA encompassing physical activity and proper dietary choices is imperative because of a significantly reduced access to healthcare globally as a result of the COVID-19 pandemic.

## 1. Introduction

Diabetes mellitus (DM) is an increasingly common metabolic disorder that is a major cause of morbidity and mortality across the globe [1]. The concept of DM self-care has gained increased attention from clinicians and researchers in the past few decades. Nevertheless, the focus has been on the discovery of novel therapies for the treatment of hyperglycaemia. However, given the fact that DM complications remain highly prevalent, increased importance has recently been given to enhancing DM education, especially through the implementation of structured education programmes. Since daily self-care activities (SCA) in DM are largely the responsibility of the patients and their families, it is expected that effective DM self-management education (DSME) will result in superior performance in the former [2]. Self-care in DM refers to the real-life application of the knowledge that the patient gained during the education programmes [3]. The goals are to bring about changes in the patient’s behaviour, thus improving glycaemic control [3].

Important components of diabetes SCA include following a healthy diet, regular physical activity, monitoring blood glucose, adherence to DM treatment, and having healthy coping and problem-solving skills related to DM activities [4]. In a study assessing the impact of DSME on glucose control, the number of patients with a HbA1c ≤ 7% increased from 27% to 70% of patients after the intervention [5]. The impact of DSME programmes appears to be significant; data from a systematic review indicate that these programmes are efficient in improving glycaemic control, reducing body mass index, lowering blood lipids, and modestly reducing blood pressure [6]. The 16-question Diabetes Self-Management Questionnaire (DSMQ), intended to be completed by patients, is a validated and useful instrument for the assessment of self-care activities [7]. Therefore, the goal of our study was to assess the level and impact of self-management activities scores in patients with DM.

## 2. Materials and Methods

The study was conducted at The National Institute of Diabetes, Nutrition and Metabolic Diseases “Prof N. C. Paulescu”, Bucharest, Romania, and the Diabetes Mellitus Clinic of the Clinical County Emergency Hospital Oradea, Romania between January and March 2020. Patients above 18 years of age with previously diagnosed DM were eligible for the study. Written informed consent was obtained from all study participants after a comprehensive explanatory discussion regarding the aims and research protocols [8]. We included adult patients between 18 and 80 years of age with a diagnosis of DM type 1 or type 2. They had to be treated with oral antidiabetic agents or injectable therapies, including long-acting insulin, rapid-acting insulin and glucagon-like peptide-1 (GLP-1) receptor agonists. Exclusion criteria included comorbidities that significantly impact glucose control, such as liver cirrhosis, end-stage kidney disease (glomerular filtration rate < 15 mL/min/1.73 m^2^), endocrine disorders (uncontrolled hypothyroidism, hyperthyroidism or Cushing disease), oncologic diseases and the presence of conditions that did not allow participants to complete the multiple-choice questionnaire (such as illiteracy, psychotic disorders, dementia, Alzheimer’s disease or advanced ophthalmologic disease).

We used the following sampling approach: every weekday during the above-specified interval, the first two patients admitted for daily hospitalisation in the National Institute of Diabetes, Nutrition and Metabolic Diseases “Prof N. C. Paulescu” and in the Diabetes Clinic of the Clinical County Emergency Hospital of Oradea were considered for inclusion in the study. Daily hospitalisation is a form of outpatient presentation through which clinical and paraclinical evaluation is performed in a 6–12 h interval (cardiology, neurology and ophthalmology consultations) every 6 months for patients with DM. A total of 159 patients were eligible after applying the inclusion and exclusion criteria; 80.5% of them had secondary education while the others (19,5%) had received higher education. The study was realised in agreement with the Clinical County Hospital of Oradea Ethical Commission nr. 25677/24.10.2019, and the research was conducted according to the principles of the Helsinki Declaration.

Patients were evaluated by history and physical examination. Body mass index (BMI) was calculated as the ratio of weight/height^2^ (kg/m^2^); systolic blood pressure (SBP) and diastolic blood pressure (DBP) were measured; the patients were evaluated for the presence of DM complications and comorbidities specified in the exclusion criteria. The following biochemical blood tests were performed: total cholesterol, low-density lipoprotein cholesterol (LDL-cholesterol), high-density lipoprotein cholesterol (HDL-cholesterol), triglycerides, fasting glycaemia, and HbA1c. The determination of HbA1c was performed using the high-performance liquid chromatography assay (HPLC) method. A desirable glucose control was defined as HbA1c < 7% (53 mmol/mol), while a suboptimal glucose control was defined as HbA1c ≥ 7% (53 mmol/mol).

All 159 patients were given information on, and assessed by, the DSMQ questionnaire, which was translated into the Romanian language. The DSMQ consisted of 16 questions with a total score (“Sum Score”). The questions were grouped into 4 subscale scores—“Glucose Management” (five questions), “Dietary Control” (four questions), “Physical Activity” (three questions), “Healthcare Use” (three questions)—and addressed the different aspects of DM self-management; there was also one overall rating question. For each completed questionnaire, the Sum Score and the subscale scores were calculated according to the authors’ instructions [7,9]. For each question, there were four possible answers ranging from 0 to 3. Each subscale score was computed as the raw score/theoretical maximum score multiplied by 10, and the subsequent total sum calculated.

Statistical analysis was performed using the Biostat software 5.9.8.5 version; the comparison of frequencies was made using the Chi-square test. In contrast, the comparison of the group mean of interval ratio scale variables was made using ANOVA. Univariate and multivariate linear regression were performed for the results of the questionnaires (the subscale scores and Sum Score) and the HbA1c level, in order to test if there was any correlation between the general level of DM self-management activities and/or the glycaemic control as evaluated by the HbA1c. A value of *p* < 0.05 was considered as statistically significant. Stepwise regression was applied by continuous elimination variables from the multivariate model in a successive manner until all the independent variables had regression coefficients different from zero and the *p*-values were less than 0.05.

## 3. Results

Patient characteristics in the groups with a HbA1c above and below 7% (53 mmol/mol) are shown in Table 1. In the univariate linear regression models (Table 2), a statistically significant inverse association was observed between the HbA1c (the dependent variable) and each DSMQ Dietary Control Score (*p* = 0.0145), DSMQ Physical Activity Score (*p* = 0.0468), and DSMQ Sum Score (*p* = 0.0014). However, as shown by the values of R2 (0.037, 0.025, and 0.06, respectively), the correlations were weak (|R| < 0.25). Since R2 (the coefficient of determination) could be interpreted as a measure of the proportion of the variance of the dependent variable explained by the model, the DSMQ Dietary Control, DSMQ Physical Activity, and DSMQ Sum Scores likely explained only small proportions of the variance of HbA1c.

Table 3 shows the full multivariate linear regression model with HbA1c as the dependent variable, and the multiple independent variables that were previously (in univariate regression models, Table 1) found to be associated with HbA1c. Table 4 shows the final, compacted multivariate linear regression model, after stepwise elimination of the independent variables found not to be associated with the dependent variable (HbA1c). The compacted model demonstrated that a greater proportion of HbA1c variance is explained when insulin resistance and insulin treatment were considered. The relative importance of the DSMQ score as a predictor of the HbA1c is small compared with these co-factors. The mean absolute change in HbA1c% associated with one standard deviation (SD) change in the DSMQ Sum Score, independent of the other significant variables retained in the compacted multivariate regression model, was −0.419 (confidence interval (CI): 95%: from −0.18 to −0.65).

## 4. Discussion

Data from the literature show that DM self-management is more effective when the patients receive in-person explanations and demonstrations. Didactic teaching courses are not as effective [10]. This process is a laborious and time-consuming one, requiring both human and financial resources. In order to analyse the impact of SCA on glucose control, we conducted a cross-sectional study to assess self-management of DM in two clinics in Romania. Results from our study revealed that an increase of one SD in the DSMQ Sum Score (representing a significant improvement in self-management behaviour) was associated with a reduction in HbA1c of 0.419%, hence the importance of an educational nutrition programme. The expected HbA1c decrease with each standard deviation increase in the DSMQ Sum Score was 0.452%. Again, this emphasises the role of all four components (nutrition, physical activity, glucose monitoring and health-care use) in achieving good DM control. In addition, a one-point decrease in the TG/HDLc ratio, indicating a significant decrease in insulin resistance, was associated with a 0.769% decrease in HbA1c. The impact of the DSMQ Score is modest when compared with the other independent variables in the multivariate model.

Although not easily achieved, behavioural modifications through structured DM education, in addition to therapeutic interventions, are of central importance in achieving good glycemic control. One example of an effective DM education programme in China consisted of theoretical and practical courses [11]. There is evidence that a number of metabolic and congenital disorders can be appropriately managed by dietary and pharmacological approaches [12,13]. In our cross-sectional analysis we did not evaluate food intake at baseline or thereafter, and this could be an important contributor to the variance. In the univariate linear regression model, the DSMQ Diet Control Score explained only 3.7% of the variance. It would be useful to use the DSMQ tool in relation to dietary assessment before and after an educational programme.

SMBG aims to enhance patients’ ability to measure the fasting, pre-meal, and postprandial glycaemia, to adjust the insulin dose according to the measured values, to recognise and prevent hyperglycaemia, and to increase the frequency of glucose monitoring during physical activity or acute illness [3]. Of the participants in the suboptimal glycaemic control group in our study, 4.76% were treated with GLP-1 receptor agonists, 63.1% with basal insulins, and 30.71% with rapid-acting insulins. The percentage of insulin-treated patients was significantly higher in patients with suboptimal vs. desirable control. Numerous studies confirm that SMBG education is effective in improving glucose control; hence the assessment of its quality must be the first step in improving it [14,15,16]. However, in our study, the DSMQ Glucose Management Score had an insignificant impact on the HbA1c, with a coefficient of determination of 0.008 with the univariate model.

This could be explained by the relatively small number of participants overall, with a higher percentage of patients in the desirable control group being treated with oral antidiabetics and measuring the capillary glucose less often because of the non-reimbursement of test strips. Schwedes et al. reported that a DM education programme that included SMBG counselling and dietary advice over a period six months (with six points-glucose self-monitoring profiles two times per week in the intensive arm of the study vs. non-standardised advice in the control arm) led to a significant improvement in HbA1c (−1% vs. −0.5%, *p* < 0.01) in the intensive arm [17]. This confirms that structured DM education is an effective intervention. Numerous high-quality studies conducted in the past decade have provided strong evidence that SBMG successfully achieves glycaemic goals [3]. Complementary measures such as reducing symptoms of depression and anxiety that are highly prevalent among DM patients were reported to increase patients’ well-being [18].

The presence of diabetic complications such as neuropathy was associated with a decreased quality of adherence using the Summary of Diabetes Self-Care Activities (SDSCA) questionnaire [19]. It also correlated with symptoms of depression [19]. In our population, 54.76 % had neuropathy, and 33.33% were diagnosed with retinopathy. The subclasses of the DSMQ could have different impacts on the management of DM depending on the population studied. In our analysis, only the score related to diet was significant. When comparing the groups in regard to HbA1c, there was no difference in the diet score between those with desirable and suboptimal control. The total score and the DSMQ Healthcare Use were lower in patients with suboptimal control. These same patients were significantly more likely treated with insulin. It highlights an unmet need for this category because they visit the diabetologist less often, and their glycaemic control is difficult to achieve.

Patients with uncontrolled DM (e.g., those with a HbA1c ≥ 7%) had significantly higher values of the TRIG/HDL ratio, which is known to be an indirect measure of atherogenic small, dense low-density lipoproteins (LDL) [20]. These particles have unique physico-chemical properties, metabolic behaviour and atherogenicity; indeed, in relation to larger, more buoyant counterparts, small, dense LDL have reduced affinity to the LDL receptor (therefore, remaining for a longer time in plasma), greater arterial entry and retention (therefore, accumulating in the intima), as well as greater susceptibility to oxidation (therefore, quickly transforming into oxidised LDL inside the artery) [21,22]. This represents the first step of the atherosclerotic process, which is enhanced in presence of endothelial dysfunction and inflammation [23,24].

It is, therefore, not surprising that at multivariate analysis, the TRIG/HDL ratio was significantly associated with the DSMQ SUM SCORE and the HbA1c. This highlights the clinical significance of atherogenic small, dense LDL in diabetic patients [25], as well as the importance of prioritising DM self-management activities in order to avoid future cardiovascular complications. Notably, insulin resistance evaluated by a TRIG/HDL ratio above 3.5 influences both the DSMQ results and the HbA1c, as we stated before. Insulin resistance was higher in sedentary patients, and the DSMQ component related to activity became more important. The activity score was not associated with the HbA1c. This could be explained by the fact that patients with desirable control were older, living in an urban environment, and probably more sedentary (for example, in quarantine). They generally have better access to healthcare facilities and information than those in rural areas—hence the elevated specific score.

The DSMQ Sum Score was significantly higher in the group with optimal control, but there are other important cofactors beside the TG/HDLc ratio, DM type and insulin treatment that were not included by us, and could explain the remaining proportion of variance in the HbA1c. Among them could be the enrolment period, which partially overlapped with the SARS-CoV-2 pandemic. Diabetic patients were considered a vulnerable group, especially during the early phase of the pandemic, when non-emergent consultations were rescheduled [26,27]. Recent studies have shown that DM self-management was challenging during the COVID-19 pandemic due to both limited access to specialised medical care and changes in lifestyle related to restrictions, social distancing, quarantine and lockdown. These probably led to a worsening of glucose control in our population. These studies have confirmed the importance of patient education for self-care, social support and a collaborative, patient-centred approach, especially in a critical time [28,29,30]. Another factor could be the participation of patients in education programmes prior to completing the questionnaire.

Our study’s main limitation consisted of the relatively small number of patients; however, we argue that systematic sampling and the application of rigorous inclusion and exclusion criteria contributed to the validity of our results. Its cross-sectional design does not provide evidence for a cause–effect relationship, but we found a negative association between the HbA1c and the DSMQ score. Prospective cohort studies and randomised controlled trials are needed to further investigate this relationship. This study brings novel information about the DM self-care in the Romanian population by showing for the first time the validity of the DSMQ translated version. Previous studies, including one with a similar population, used different questionnaires to assess the quality of adherence [19]. In the study validating the questionnaire, Schmitt et al. [9] specified a correlation coefficient in the total sample of −0.40 between the HbA1c and the DSMQ Sum Score. For type 2 diabetic patients, like most subjects in our sample, the coefficient was −0.38. This value is slightly larger than the one we found (R= −0.24), suggesting that the Romanian-language version has a similar predictive validity.

In summary, findings from our study confirmed that adherence to self-care activities significantly influenced glycaemic control. Our work complements the results of other studies realised in different cultural areas of Europe [15,31,32], and argues that the DSMQ is a useful tool for analysing behaviour related to HbA1c, namely glycaemic control [15]. We wish to highlight the importance of educational programmes, intensive physical activity, dietary regimens, comprehensive lifestyle intervention and treatments in diabetic patients; the efficacy of lifestyle interventions in patients with type 2 diabetes has been investigated in a systematic review and meta-analysis, showing that nutritional intervention had a significant impact on the quality of life of these patients, by reducing their cardiovascular risk [33]. Since this study was performed during the very early phase of the COVID-19 pandemic in Romania, it emphasises the importance of optimal management of DM to avoid uncontrolled hyperglycaemia and future complications [34]. The COVID-19 pandemic has had a major impact on Romanian society [35]; this has important implications for patients with DM, because the DSMQ was negatively influenced by the pandemic in other studies [36]. Patients with DM are among the most vulnerable to severity and mortality from COVID-19 [37] and are experiencing challenges in accessing healthcare facilities and clinic visits; this is contributing to the increase in their cardiometabolic complications [38]. The role of the SCA and the DSMQ assessment in achieving tight glycaemic control under these circumstances is, therefore, of increased significance.

## 5. Conclusions

Enhanced self-management of DM is associated with improved glucose control. Since effective self-management ensues in large part from optimising DM education, clinicians should focus on improving patients’ knowledge and self-efficacy regarding nutrition, physical activity, glucose self-monitoring, and use of healthcare technology, all covered by the DSMQ tool.

## Figures and Tables

**Table 1:** Characteristics of the included patients.

Parameter	Unit of Measure	HbA1c <7 (*n* = 75)	HbA1c ≥ 7 (*n* = 84)	*p*-Value
Sex (men)	%	50.00	50.67	0.97
Age	Years	64.48 ± 8.43	59.14 ± 12.38	<0.01 *
Living Environment (urban)	%	76.00	58.33	0.01 *
Type 2 diabetes mellitus	%	91.66	100.00	0.87
BMI	kg/m^2^	29.76 ± 5.68	30.45 ± 7.33	0.50
HbA1c	%	6.27 ± 0.57	8.87 ± 1.60	<0.01 *
DSMQ SUM Score (max = 10)	Points	6.99 ± 1.42	6.32 ± 1.54	<0.01 *
DSMQ HEALTHCARE USE Score (max = 10)	Points	7.54 ± 1.56	6.98 ± 1.87	0.04 *
DSMQ PHYSICAL ACTIVITY Score (max = 10)	Points	5.86 ± 3.45	5.36 ± 3.16	0.34
DSMQ DIETARY CONTROL Score (max = 10)	Points	6.39 ± 2.69	5.68 ± 2.26	0.07
DSMQ GLUCOSE MANAGEMENT Score (max = 10)	Points	7.28 ± 1.29	6.91 ± 1.81	0.13
Diabetic neuropathy	%	42.67	54.76	0.12
Diabetic retinopathy	%	18.67	33.33	0.03 *
Diabetic nephropathy	%	18.67	30.95	0.07
Cardiovascular disease	%	23.81	25.33	0.82
Ischemic heart disease	%	25.33	32.14	0.34
Hypertension	%	66.67	89.33	<0.01 *
Heart failure	%	10.67	14.29	0.45
LDL-cholesterol	mg/dL	102.52 ± 45.19	115.22 ± 51.97	0.10
Triglycerides (Q1, Q3)	mg/dL	79.75 (68.5, 98.7)	111.5 (90.2, 134.8)	<0.01 *
HDL-cholesterol	mg/dL	44.72 ± 13.07	44.52 ± 11.03	0.91
TRIG/HDL		3.28 ± 1.87	4.23 ± 4.0	0.01 *
OAD	%	78.67	61.90	0.02 *
GLP-1 receptor agonists	%	6.67	4.76	0.60
Long-acting Insulin	%	24.00	63.10	<0.01 *
Rapid-acting Insulin	%	6.67	30.71	<0.01 *

* Statistically significant; Data are expressed as mean ± standard deviation for normally distributed data, and median (IQ range) for non-normally distributed data. BMI—body mass index; LDL-cholesterol—low-density lipoprotein cholesterol; TRIG—triglycerides; HDL-cholesterol—high-density lipoprotein cholesterol; TRIG/HDL—triglycerides/high-density lipoprotein; OAD—oral antidiabetic drugs; GLP-1 receptor agonists—glucagon-like peptide-1 receptor agonists.

**Table 2:** Parameters of univariate linear regression between DSMQ questionnaire components and HbA1c.

Independent Variable	B	Standard Error	LCL	UCL	*p*-Value	R^2^
DSMQ Glucose Management Score	−0.09	0.08	−0.24	0.06	0.2451	0.008
DSMQ Dietary Control Score	−0.14	0.06	−0.25	−0.03	0.0145 *	0.037
DSMQ Physical Activity Score	−0.09	0.04	−0.17	0.00	0.0468	0.025
DSMQ Healthcare Use	−0.06	0.08	−0.22	0.10	0.4677	0.003
DSMQ SUM SCORE	−0.30	0.09	−0.48	−0.12	0.0014 *	0.06

* Statistically significant; LCL—lower confidence limit; UCL—upper confidence limit; DSMQ—Diabetes Self-Management Questionnaire.

**Table 3:** Multivariate linear regression model with HbA1c as the dependent variable, and multiple independent variables.

Independent Variable	B	Standard Error	LCL	UCL	*p*-Value
DSMQ SUM SCORE (max = 10)	−0.32	0.08	−0.48	−0.16	0.0001 *
Sex	0.34	0.26	−0.17	0.85	0.1845
Age	0.01	0.01	−0.01	0.03	0.4378
Living environment	−0.53	0.26	−1.05	−0.01	0.0464
Type of diabetes mellitus	−1.65	0.66	−2.95	−0.36	0.0129 *
Obesity	0.17	0.26	−0.33	0.67	0.5044
TRIG/HDL-cholesterol ratio >= 3.5	0.87	0.24	0.40	1.35	0.0004 *
Oral antidiabetic drugs	0.61	0.35	−0.09	1.31	0.0861
GLP-1 receptor agonists treatment	0.09	0.53	−0.97	1.14	0.8682
Long-acting insulin	0.88	0.31	0.27	1.50	0.0053 *
Rapid-acting insulin	1.62	0.40	0.83	2.41	0.0001 *

* Statistically significant; DSMQ—Diabetes Self-Management Questionnaire; LCL—lower confidence limit; UCL—upper confidence limit; TRIG/HDL—triglycerides/high-density lipoprotein; GLP-1—glucagon-like peptide-1. Obesity is defined as BMI ≥ 30kg/m^2^.

**Table 4:** Correlation between DSMQ Sum Score and HbA1c in a multivariate model, after step-wise elimination of variables.

Independent Variable	B	Standard Error	LCL	UCL	*p*-Value
DSMQ SUM SCORE (max = 10)	−0.2835	0.0789	−0.4394	−0.1275	0.0004
Type of diabetes mellitus	−1.3355	0.6374	−2.5948	−0.0762	0.0378
TRIG/HDL-cholesterol ratio >= 3.5	0.7690	0.2409	0.2932	1.2449	0.0017
Long-acting insulin	0.7009	0.2949	0.1184	1.2835	0.0187
Rapid-acting insulin	1.2528	0.3764	0.5092	1.9964	0.0011

* Statistically significant; DSMQ—Diabetes Self-Management Questionnaire; LCL—lower confidence limit; UCL—upper confidence limit; TRIG/HDL—triglycerides/high-density lipoprotein.

## Data Availability

Data are available on request with the permission of C.M.V.
